# Evolution of codon usage in *Taenia saginata* genomes and its impact on the host

**DOI:** 10.3389/fvets.2022.1021440

**Published:** 2023-01-11

**Authors:** Siddiq Ur Rahman, Hassan Ur Rehman, Inayat Ur Rahman, Muazzam Ali Khan, Fazli Rahim, Hamid Ali, Dekun Chen, Wentao Ma

**Affiliations:** ^1^Department of Computer Science and Bioinformatics, Khushal Khan Khattak University, Karak, Pakistan; ^2^Department of Botany, Khushal Khan Khattak University, Karak, Pakistan; ^3^Department of Botany, Bacha Khan University, Charsadda, KP, Pakistan; ^4^Department of Biotechnology and Genetic Engineering, Hazara University, Mansehra, Pakistan; ^5^College of Veterinary Medicine, Northwest A&F University, Yangling, Shaanxi, China; ^6^Veterinary Immunology Laboratory, College of Veterinary Medicine, Northwest A&F University, Yangling, Shaanxi, China

**Keywords:** *Taenia saginata*, codon usage bias (CUB), effective number of codons (ENC), mutation pressure, natural selection

## Abstract

The beef tapeworm, also known as *Taenia saginata*, is a zoonotic tapeworm from the genus *Taenia* in the order Cyclophyllidea. *Taenia saginata* is a food-borne zoonotic parasite with a worldwide distribution. It poses serious health risks to the host and has a considerable negative socioeconomic impact. Previous studies have explained the population structure of *T. saginata* within the evolutionary time scale and adaptive evolution. However, it is still unknown how synonymous codons are used by *T. saginata*. In this study, we used 90 *T. saginata* strains, applying the codon usage bias (CUB). Both base content and relative synonymous codon usage (RSCU) analysis revealed that AT-ended codons were more frequently used in the genome of *T. saginata*. Further low CUB was observed from the effective number of codons (ENC) value. The neutrality plot analysis suggested that the dominant factor of natural selection was involved in the structuring of CUB in *T. saginata*. Further analysis showed that *T. saginata* has adapted host-specific codon usage patterns to sustain successful replication and transmission chains within hosts (*Bos taurus* and *Homo sapiens*). Generally, both natural selection and mutational pressure have an impact on the codon usage patterns of the protein-coding genes in *T. saginata*. This study is important because it characterized the codon usage pattern in the *T. saginata* genomes and provided the necessary data for a basic evolutionary study on them.

## 1. Introduction

Codon usage bias (CUB) refers to the phenomenon in which synonymous codons are not used with equal frequency during gene translation. CUB is a common phenomenon in numerous species, including prokaryotes and eukaryotes (1, 2). It has been noted that a variety of factors affect how codons are used by different organisms. The primary explanations for the variance in codon usage among the genes in these species are assumed to be weak natural selection and mutational pressure (3). Understanding the fundamental characteristics of a genome's molecular organization requires extensive research into codon usage patterns across the entire genome. Furthermore, analysis of CUB has numerous other crucial applied aspects, including heterologous gene expression (4), identifying species origins (5), designing degenerate primers (6), predicting gene expression levels (7, 8), and predicting gene functions (9). However, the majority of the numerous reports on CUB have concentrated on model organisms and numerous microorganisms, including yeast (10), *Giardia lamblia* (11), and *Entamoeba histolytica* (12). For instance, it has been noted that most preferred codons in *Caenorhabditis elegans* end in G or C (the majority being C endings) (13). In contrast, studies on tapeworms are limited. *Taenia saginata* is a significant parasitic tapeworm with a large geographical distribution (14, 15).

The beef tapeworm, also known as *T. saginata*, is a zoonotic tapeworm from the genus *Taenia* in the order Cyclophyllidea (16). It is the most frequent tapeworm that causes taeniasis in humans and cysticercosis in cattle (17). Additionally, in Europe, the infection has been found in both cattle and humans, indicating that the parasite is continuously spreading (18, 19). *Taenia saginata* is found in all continents and is endemic to eastern Europe, Southeast Asia, Africa, and Latin America (20–22). However, in addition to the classic strain of this parasite found in southern regions, which is associated with cattle raising, there is a lesser known form of *T. saginata* in northern regions (20). *Taenia saginata* produces long-term infections, being able to survive within its host for several years, maintaining a dynamic and complex host-parasite relationship (23, 24). *Taenia saginata* lives in cattle as an intermediate host while in humans as a definitive host (15). Their eggs passed in the feces of an infected person are only infectious to cattle. Taeniasis, or human tapeworm infection, is induced by eating undercooked contaminated meat and usually results in very mild clinical symptoms (14, 17), with few complications, such as an intestinal blockage. Most patients infected with *T. saginata* have epigastric pain, nausea, weight loss, and poor appetite (25). Cattle infected with *T. saginata* have a significant negative impact on the economy in terms of food production and trade restrictions (26). Uncertainty exists about the pattern of synonymous codon usage by *T. saginata*. In this study, we used a multivariate statistical analysis to examine the codon usage patterns of *T. saginata* using complete coding data. Understanding the underlying mechanism for biased usage of synonymous codons and selecting suitable host expression systems for optimal expression of target genes would be made possible by analysis of the codon usage pattern in *T. saginata*.

## 2. Materials and methods

### 2.1. Sequences

A total of 90 complete coding sequences (CDSs) of the *T. saginata* were retrieved from the National Center for Biotechnology Information (NCBI) GenBank database (https://ncbi.nlm.nih.gov/nuccore/?term=Taenia+saginata). The number of nucleotides in the coding sequence was an exact multiple of three (27). Complete information about the overall 90 *T. saginata* strains associated with Asian, African, and European countries is listed in Supplementary Table S1.

### 2.2. Analysis of nucleotide composition

In this study, we employed CodonW software to determine the total base composition (G, C, A, and T%) and the contents of the nucleotide at the 3rd codon location (C3, T3, G3, and A3%) for all synonymous codons in *T. saginata*. The GC% contents of all three codon locations (GC1, GC2, and GC3%) were measured. Additionally, the average frequency of G/C at GC12 locations and the overall GC/AT compositions were also determined. Furthermore, only 59 synonymous codons encoding 18 amino acids were considered for the present study, not including the first ATG codon, the codon (TGG) encoding tryptophan, and the three termination codons (TAG, TAA, and TGA), respectively (5).

### 2.3. Analysis of relative synonymous codon usage

Relative synonymous codon usage (RSCU) values indicate the observed codon occurrence to its random occurrence, suggesting that all the identical codons of the *T. saginata* are equal in usage. There are frequently occurring codons that have an RSCU value greater than one, and less frequently occurring codons that have an RSCU value less than one in the CDS (28). Higher CUB or more frequently used codons were determined through high RSCU. In the coding sequence, the overrepresented codon represents the codon RSCU value >1.6, and the underrepresented codon represents the codon RSCU value <0.6 (29). The RSCU value was determined for each codon using the following formula (30):


$$\begin{array}{l} RSCU=\frac{X_{ij}}{\sum_{j=1}^{n_{i}}X_{ij}}n_{i}. \end{array}$$

In this study, *n*_*i*_ is the number of codons for the *i*th amino acid, and *X*_*ij*_ denotes the frequency of the *j*th codon for the *i*th amino acid.

### 2.4. Analysis of codon adaptation index

The codon adaptation index (CAI) is applied to calculate the gene expression level depending on its codon-based sequence. The value of CAI value varies from zero to one; a value near one indicates higher levels of CUB (31). The CAI was determined through an online tool used for CAI calculation, “CAIcal” (32) where the *Bos taurus* and *Homo sapiens* genomes were used as a reference source. Furthermore, the e-CAI (expected CAI) was analyzed using the online tool “CAIcal.” The values of RSCU for the *B. taurus* and *H. sapiens* genomes were retrieved from the database of codon usage.

### 2.5. Similarity index analysis

The similarity index (SiD) measuring how the overall codon usage pattern of the host affects the overall codon usage of the parasite was determined as follows:


$$\begin{array}{l} R\left(A,B\right)=\;\frac{\sum_{i=1}^{59}a_{i}\times b_{i}}{\sqrt{\sum_{i=1}^{59}a_{i}^{2}\times \sum_{i=1}^{59}b_{i}^{2}}} \end{array}$$

$$\begin{array}{l} D\left(A,B\right)=\frac{1-R\left(A,B\right)}{2} \end{array}$$

where *R*(A, B) denotes the degree of similarity between the host and the *T. saginata* overall codon usage patterns, which is defined as the cosine value of the included angle between A and B. Among the 59 synonymous codons in *T. saginata*, *a*_i_ is defined as the RSCU value for a particular codon. The RSCU value for the host's identical codon is known as *b*_i_. The value of *D*(A, B) ranges from 0 to 1.0 and shows the potential impact of the host's total codon usage on that of *T. saginata* (33).

### 2.6. Indices of codon usage

To determine the proper measurement of codon bias, ENC (effective number of codons) was performed, which measures the total usage of the codon in a certain gene (34, 35). It clarifies the ratio of codon variation in a gene from the total even usage of codons, which are synonymous. The ENC value varies from 20 (where one amino acid encodes one codon only) to 61 (where each amino acid is used randomly for all codons). ENC value <35 implies significant CUB (34, 36). A plot between ENC-GC3s is drawn out to determine the impact of GC3s composition on codon usage (34). For each GC3, the expected ENC values were considered using the following formula:


$$\begin{array}{l} ENC=2+s+\frac{29}{\left(s^{2}+{\left(1-s\right)}^{2}\right)} \end{array}$$

where “s” is the GC3s content of each codon.

### 2.7. Correspondence analysis

Correspondence analysis (COA) is a multidimensional critical method to resolve the important developments in the codon usage patterns of CDS through codon RSCU values (5, 37, 38). To create the COA plot, RSCU values of 59 codons were considered. To study the tendencies in the deviation of the use of codon, relative inertia was used to hold a specific position in the graph.

### 2.8. Phylogenetic analysis of *Taenia saginata*

The phylogenetic tree was constructed using the maximum likelihood method in Clustal ×2 (http://www.clustal.org/clustal2/). The tree was designed using the online tool the Interactive Tree of Life version 3 (http://itol.embl.de/) (39, 40). A total of 90 strains were used in this study.

### 2.9. Analysis of neutrality plot

GC12 and GC3 were studied for attaining a neutrality plot to identify the role of selection-mutation symmetry in the usage of codon discrepancy. In the graph, each point signifies a discrete gene. The line of regression slop between GC3 and GC12 indicates that mutation pressure is the major factor in affecting CUB, i.e., for values coming close to 1, although if the value comes close to 0, it indicates that selection pressure has been the main factor in defining CUB (41, 42).

### 2.10. Correlation analysis

To illustrate the relationship between nucleotide content and codon usage patterns, *T. saginata* correlation analysis was performed. These analyses were conducted using Spearman's rank correlation method (43). All processes were executed using the R corrplot package. For codon usage index analysis, CodonW (1.4.4) software was applied (44, 45).

## 3. Results and discussion

### 3.1. Basic compositional analysis in *Taenia saginata* coding sequences

**Figure 1:**
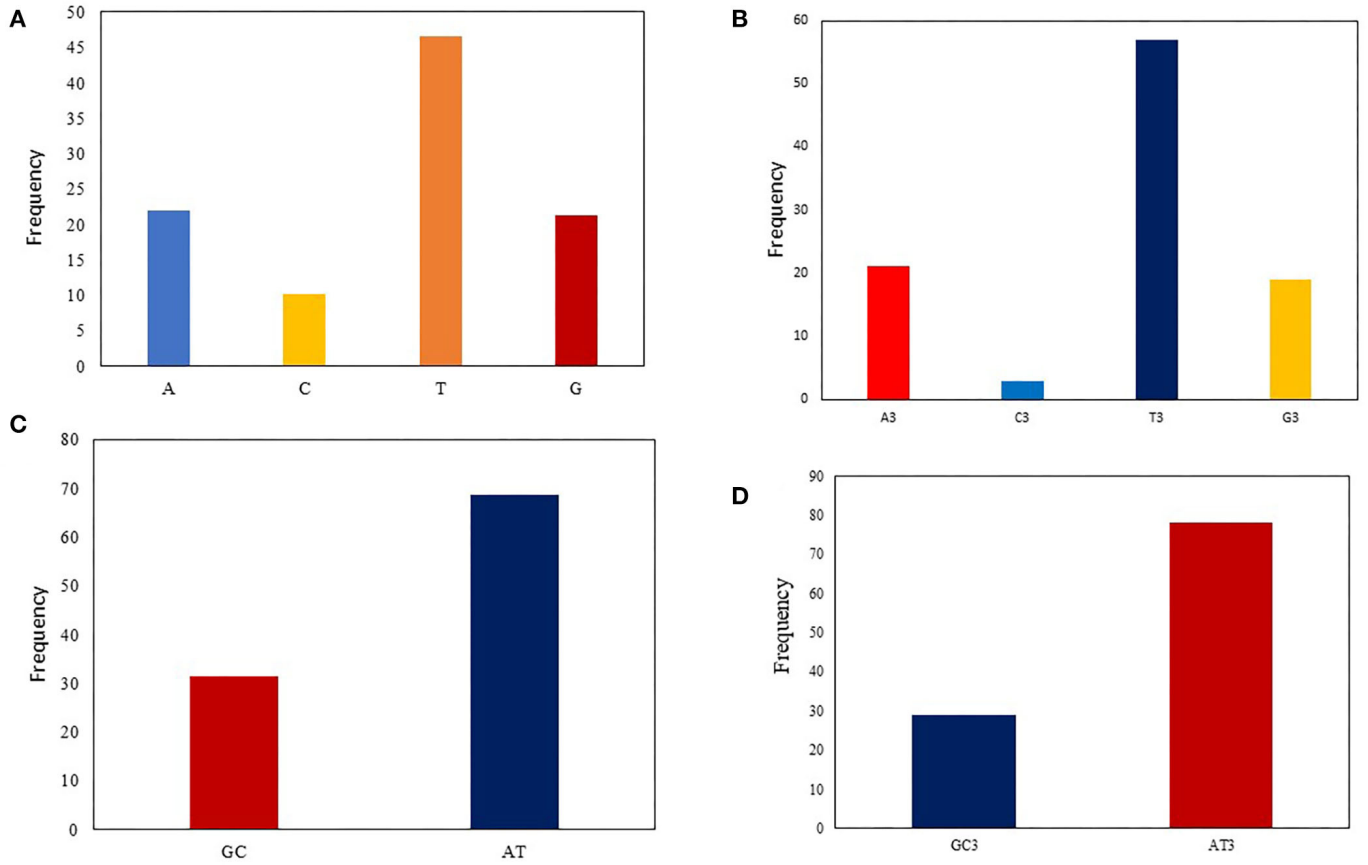
Nucleotide composition analysis: **(A)** The average A, T, G, and C nucleotide composition of the entire viral genome. **(B)** The average values of the nucleotide composition at the third codon position, indicating A/T richness followed by G/C richness. **(C)** The mean frequency for GC and AT composition **(D)** The mean frequency of GC and AT at the codon's third position, indicating that AT3 is more common than GC3.

Codon usage bias can be considerably predisposed by the general base composition of genomes. The nucleotide contents of 90 *T. saginata* strains were studied, which are presented in Table 1. In this study, our outcomes disclosed that the mean A (21.9%) and T (46.6%) were maximum, tailed by G (21.3%) and C (10.1%) across all genomes. The mean A3 (21.15%) and T3 (56.96%) occurred at a maximum level higher than the G3 (18.96%) and C3 (2.93%) (Figure 1, Table 1). The total AT and GC compositions were found to be 68.54% and 31.46%, respectively, suggesting that *T. saginata* strains have strong AT. This finding is similar to previous research on *Plasmodium falciparum, Mycoplasma capricolum*, and *Onchocerca volvulus* being enriched with A and T (46). However, the biological significance of this condition is still unclear, and therefore, it is essential to explore the causes for increased AT contents and decreased GC contents in the parasite genomes (47).

**Table 1:** Nucleotide compositional analysis of *Taenia saginata* coding sequences (%).

**Sequences**	**A**	**C**	**T**	**G**	**GC**	**AT**	**GC1**	**GC2**	**GC3**	**A3**	**C3**	**T3**	**G3**	**GC3**	**AT3**	**ENC**
AB984346.1	21.67	9.81	47.28	21.23	31.05	68.95	34.26	38.52	20.37	20.93	1.67	58.70	18.70	20.37	79.63	33.7
AB984347.1	21.54	9.88	47.35	21.23	31.11	68.89	34.26	38.33	20.74	20.74	1.85	58.52	18.89	20.74	79.26	33.4
AB984348.1	21.54	9.75	47.35	21.36	31.11	68.89	34.07	38.52	20.74	20.56	1.67	58.70	19.07	20.74	79.26	33.8
AB984349.1	21.73	9.75	47.35	21.17	30.93	69.07	34.07	38.33	20.37	20.93	1.67	58.70	18.70	20.37	79.63	33.8
AB984350.1	21.60	9.69	47.41	21.30	30.99	69.01	33.89	38.33	20.74	20.56	1.67	58.70	19.07	20.74	79.26	33.8
AB984351.1	21.67	9.81	47.28	21.23	31.05	68.95	34.07	38.33	20.74	20.74	1.85	58.52	18.89	20.74	79.26	34.2
AB984352.1	21.48	9.57	47.41	21.54	31.11	68.89	33.89	37.96	21.48	20.37	1.85	58.15	19.63	21.48	78.52	34.5
AB533168.1	21.67	9.75	47.35	21.23	30.99	69.01	34.07	38.33	20.56	20.74	1.67	58.70	18.89	20.56	79.44	33.8
AB533169.1	21.52	9.77	47.43	21.27	31.05	68.95	36.73	23.38	33.02	25.23	8.72	41.74	24.30	33.02	66.98	42.9
AB533171.1	21.73	9.75	47.35	21.17	30.93	69.07	34.07	38.33	20.37	20.93	1.67	58.70	18.70	20.37	79.63	33.8
AB533172.1	21.73	9.69	47.41	21.17	30.86	69.14	33.89	38.52	20.19	21.11	1.67	58.70	18.52	20.19	79.81	33.7
MN452861.1	21.93	5.70	50.11	22.26	27.96	72.04	28.29	35.86	19.74	22.37	0.99	57.89	18.75	19.74	80.26	35.6
MN452862.1	21.73	9.63	47.47	21.17	30.80	69.20	34.07	38.33	20.00	21.11	1.48	58.89	18.52	20.00	80.00	33.6
AB066495.1	21.67	9.75	47.28	21.30	31.05	68.95	34.07	38.15	20.93	20.56	1.85	58.52	19.07	20.93	79.07	34
AB066581.1	23.03	8.52	47.38	21.07	29.59	70.41	35.11	32.30	21.35	23.31	1.12	55.34	20.22	21.35	78.65	36.3
AB107239.1	21.60	9.69	47.41	21.30	30.99	69.01	34.07	38.52	20.37	20.74	1.48	58.89	18.89	20.37	79.63	33.6
AB465247.1	21.60	9.75	47.35	21.30	31.05	68.95	34.07	38.52	20.56	20.74	1.67	58.70	18.89	20.56	79.44	33.8
JX489220.1	25.99	8.01	28.71	22.40	45.31	54.69	46.18	42.96	46.78	26.06	1.10	27.16	23.44	46.78	53.22	58.3
KJ941091.1	29.58	8.5	23.76	24.85	46.67	53.33	47.64	40.73	51.64	22.55	1.31	25.82	24.36	51.64	48.36	55.9
MK644930.1	21.67	9.75	47.35	21.23	30.99	69.01	34.07	38.33	20.56	20.74	1.67	58.70	18.89	20.56	79.44	33.8
MK644932.1	21.67	9.63	47.47	21.23	30.86	69.14	34.07	38.15	20.37	20.93	1.67	58.70	18.70	20.37	79.63	33.7
AY147845.1	26.13	9.2	27.69	21.55	46.17	53.83	44.43	46.77	47.32	25.31	1.46	27.37	22.70	47.32	52.68	57.3
MK644933.1	21.67	9.63	47.47	21.23	30.86	69.14	34.07	38.15	20.37	20.93	1.67	58.70	18.70	20.37	79.63	33.7
MK644934.1	21.67	9.75	47.35	21.23	30.99	69.01	34.07	38.33	20.56	20.74	1.67	58.70	18.89	20.56	79.44	33.8
AB465246.1	21.60	9.75	47.35	21.30	31.05	68.95	34.07	38.52	20.56	20.74	1.67	58.70	18.89	20.56	79.44	33.8
MK644931.1	21.67	9.63	47.47	21.23	30.86	69.14	34.07	38.15	20.37	20.93	1.67	58.70	18.70	20.37	79.63	33.7
AB465231.1	21.67	9.75	47.35	21.23	30.99	69.01	34.07	38.33	20.56	20.74	1.67	58.70	18.89	20.56	79.44	33.8
AB465232.1	21.67	9.75	47.35	21.23	30.99	69.01	34.07	38.33	20.56	20.74	1.67	58.70	18.89	20.56	79.44	33.8
AB465233.1	21.67	9.75	47.35	21.23	30.99	69.01	34.07	38.33	20.56	20.74	1.67	58.70	18.89	20.56	79.44	33.8
AB465234.1	21.67	9.75	47.35	21.23	30.99	69.01	34.07	38.33	20.56	20.74	1.67	58.70	18.89	20.56	79.44	33.8
AB465235.1	21.73	9.69	47.41	21.17	30.86	69.14	34.07	38.33	20.19	21.11	1.67	58.70	18.52	20.19	79.81	33.7
AB465236.1	21.73	9.69	47.41	21.17	30.86	69.14	34.07	38.33	20.19	21.11	1.67	58.70	18.52	20.19	79.81	33.7
AB465239.1	21.79	9.63	47.47	21.11	30.74	69.26	33.89	38.33	20.00	21.11	1.48	58.89	18.52	20.00	80.00	33.5
AB465242.1	21.60	9.75	47.35	21.30	31.05	68.95	34.07	38.33	20.74	20.56	1.67	58.70	19.07	20.74	79.26	33.8
AB465247.1	21.60	9.75	47.35	21.30	31.05	68.95	34.07	38.52	20.56	20.74	1.67	58.70	18.89	20.56	79.44	33.8
AB465248.1	21.60	9.75	47.35	21.30	31.05	68.95	34.07	38.52	20.56	20.74	1.67	58.70	18.89	20.56	79.44	33.8
AB533173.1	21.79	9.69	47.35	21.17	30.86	69.14	34.07	38.33	20.19	21.30	1.67	58.52	18.52	20.19	79.81	33.7
KY290351.1	21.54	9.69	47.41	21.36	31.05	68.95	34.07	38.33	20.74	20.37	1.48	58.89	19.26	20.74	79.26	33.6
KY290352.1	21.48	9.63	47.47	21.42	31.05	68.95	34.26	38.52	20.37	20.56	1.30	59.07	19.07	20.37	79.63	33.3
KY290353.1	21.60	9.69	47.41	21.30	30.99	69.01	34.07	38.33	20.56	20.56	1.48	58.89	19.07	20.56	79.44	33.6
KY290354.1	21.54	9.57	47.53	21.36	30.93	69.07	33.70	38.52	20.56	20.56	1.48	58.89	19.07	20.56	79.44	33.6
KY290355.1	21.54	9.69	47.41	21.36	31.05	68.95	34.07	38.33	20.74	20.37	1.48	58.89	19.26	20.74	79.26	33.6
KY290356.1	21.54	9.63	47.47	21.36	30.99	69.01	34.07	38.52	20.37	20.56	1.30	59.07	19.07	20.37	79.63	33.3
KY290357.1	21.67	9.75	47.35	21.23	30.99	69.01	34.07	38.33	20.56	20.93	1.85	58.52	18.70	20.56	79.44	33.8
KY290358.1	21.54	9.69	47.41	21.36	31.05	68.95	34.07	38.33	20.74	20.37	1.48	58.89	19.26	20.74	79.26	33.6
AB107244.1	21.67	9.75	47.35	21.23	30.99	69.01	34.07	38.33	20.56	20.74	1.67	58.70	18.89	20.56	79.44	33.8
AB107245.1	21.73	9.69	47.41	21.17	30.86	69.14	34.07	38.33	20.19	21.11	1.67	58.70	18.52	20.19	79.81	33.7
AB107849.1	19.96	6.98	52.91	20.16	27.13	72.87	33.72	30.81	16.86	20.35	2.33	62.79	14.53	16.86	83.14	30.2
AB441816.1	27.17	7.1	31.94	21.64	40.88	59.12	41.42	41.04	40.19	25.88	1.71	33.93	22.18	40.19	59.81	56
KY290359.1	21.67	9.69	47.41	21.23	30.93	69.07	34.26	38.33	20.19	20.93	1.48	58.89	18.70	20.19	79.81	33.6
KY290360.1	21.73	9.69	47.41	21.17	30.86	69.14	34.07	38.15	20.37	20.74	1.48	58.89	18.89	20.37	79.63	33.7
KY290364.1	21.60	9.69	47.47	21.23	30.93	69.07	34.07	38.33	20.37	20.74	1.67	58.89	18.70	20.37	79.63	33.6
KY290365.1	21.73	9.69	47.41	21.17	30.86	69.14	34.07	38.33	20.19	20.93	1.48	58.89	18.70	20.19	79.81	33.6
KY290366.1	21.67	9.63	47.47	21.23	30.86	69.14	34.07	38.33	20.19	20.93	1.48	58.89	18.70	20.19	79.81	33.5
KY290367.1	21.67	9.69	47.41	21.23	30.93	69.07	34.07	38.52	20.19	20.93	1.48	58.89	18.70	20.19	79.81	33.6
KY290368.1	21.73	9.75	47.35	21.17	30.93	69.07	34.07	38.33	20.37	21.11	1.85	58.52	18.52	20.37	79.63	33.7
KY290369.1	21.73	9.69	47.41	21.17	30.86	69.14	34.07	38.33	20.19	21.11	1.67	58.70	18.52	20.19	79.81	33.6
KY290370.1	21.67	9.63	47.47	21.23	30.86	69.14	34.07	38.33	20.19	20.74	1.30	59.07	18.89	20.19	79.81	33.5
KY290371.1	21.60	9.69	47.41	21.30	30.99	69.01	34.26	38.33	20.37	20.74	1.48	58.89	18.89	20.37	79.63	33.6
KY290372.1	21.60	9.69	47.41	21.30	30.99	69.01	34.07	38.33	20.56	20.56	1.48	58.89	19.07	20.56	79.44	33.7
KY290373.1	21.67	9.69	47.41	21.23	30.93	69.07	34.26	38.33	20.19	20.93	1.48	58.89	18.70	20.19	79.81	33.6
AB465237.1	21.60	9.75	47.35	21.30	31.05	68.95	34.07	38.52	20.56	20.74	1.67	58.70	18.89	20.56	79.44	34
AB465245.1	21.60	9.75	47.35	21.30	31.05	68.95	34.07	38.52	20.56	20.74	1.67	58.70	18.89	20.56	79.44	33.8
AB107241.1	21.60	9.81	47.28	21.30	31.11	68.89	34.07	38.52	20.74	20.74	1.85	58.52	18.89	20.74	79.26	34
AB465241.1	21.73	9.75	47.35	21.17	30.93	69.07	34.07	38.33	20.37	20.93	1.67	58.70	18.70	20.37	79.63	33.8
MT074048.1	21.83	9.72	47.34	21.10	30.83	69.17	33.94	37.98	20.55	21.10	2.02	58.35	18.53	20.55	79.45	33.7
MT074049.1	21.71	9.72	47.34	21.22	30.95	69.05	33.94	38.17	20.73	20.92	2.02	58.35	18.72	20.73	79.27	33.8
MT074050.1	21.77	9.72	47.34	21.16	30.89	69.11	33.94	38.17	20.55	21.10	2.02	58.35	18.53	20.55	79.45	33.8
AB274525.1	22.85	8.61	47.38	21.16	29.78	70.22	35.67	32.30	21.35	23.31	1.12	55.34	20.22	21.35	78.65	36.6
AB275143.1	21.73	9.75	47.35	21.17	30.93	69.07	34.07	38.33	20.37	20.93	1.67	58.70	18.70	20.37	79.63	33.8
AB465238.1	21.67	9.69	47.41	21.23	30.93	69.07	34.07	38.33	20.37	20.74	1.48	58.89	18.89	20.37	79.63	33.6
AB107246.1	21.73	9.69	47.41	21.17	30.86	69.14	33.89	38.33	20.37	20.93	1.67	58.70	18.70	20.37	79.63	33.7
AB107847.1	19.96	6.78	53.10	20.16	26.94	73.06	33.72	30.81	16.28	20.35	1.74	63.37	14.53	16.28	83.72	30.1
AB465244.1	21.67	9.69	47.41	21.23	30.93	69.07	34.07	38.15	20.56	20.74	1.67	58.70	18.89	20.56	79.44	33.8
AB645845.1	21.60	9.75	47.35	21.30	31.05	68.95	34.07	38.33	20.74	20.56	1.67	58.70	19.07	20.74	79.26	34.1
AB644391.1	21.60	9.75	47.35	21.30	31.05	68.95	34.07	38.52	20.56	20.74	1.67	58.70	18.89	20.56	79.44	33.8
AB821273.1	21.60	9.81	47.28	21.30	31.11	68.89	34.07	38.52	20.74	20.74	1.85	58.52	18.89	20.74	79.26	34
AB820291.1	21.58	9.83	47.20	21.39	31.22	68.78	34.14	38.81	20.71	20.52	1.68	58.77	19.03	20.71	79.29	33.8
AB465243.1	21.60	9.69	47.41	21.30	30.99	69.01	34.07	38.33	20.56	20.74	1.67	58.70	18.89	20.56	79.44	33.8
AB107238.1	21.73	9.75	47.35	21.17	30.93	69.07	34.07	38.33	20.37	20.93	1.67	58.70	18.70	20.37	79.63	33.8
AB107850.1	19.96	6.78	53.10	20.16	26.94	73.06	33.72	30.81	16.28	20.35	1.74	63.37	14.53	16.28	83.72	30.1
AB465240.1	21.73	9.75	47.35	21.17	30.93	69.07	34.07	38.15	20.56	20.74	1.67	58.70	18.89	20.56	79.44	33.8
AB107240.1	21.60	9.75	47.35	21.30	31.05	68.95	34.07	38.52	20.56	20.74	1.67	58.70	18.89	20.56	79.44	33.8
AB107846.1	19.96	6.59	53.29	20.16	26.74	73.26	33.72	30.23	16.28	20.35	1.74	63.37	14.53	16.28	83.72	30.2
HQ318711.1	26.36	6.81	26.51	25.65	47.13	52.87	47.63	45.69	48.06	25.22	1.52	26.72	23.49	48.06	51.94	55.8
AB107242.1	21.67	9.69	47.41	21.23	30.93	69.07	34.07	38.15	20.56	20.74	1.67	58.70	18.89	20.56	79.44	33.5
AB107243.1	21.67	9.75	47.35	21.23	30.99	69.01	33.89	38.33	20.74	20.74	1.85	58.52	18.89	20.74	79.26	34
AB107848.1	20.16	6.78	53.10	19.96	26.74	73.26	33.14	30.81	16.28	20.35	1.74	63.37	14.53	16.28	83.72	30.1
AB271695.1	21.54	9.69	47.41	21.36	31.05	68.95	33.89	38.52	20.74	20.56	1.67	58.70	19.07	20.74	79.26	34
AB271696.1	22.94	8.61	47.19	21.25	29.87	70.13	35.67	32.30	21.63	23.03	1.12	55.34	20.51	21.63	78.37	36.5
**Means**	**21.90**	**10.15**	**46.64**	**21.31**	**31.46**	**68.54**	**34.68**	**37.81**	**21.89**	**21.15**	**2.93**	**56.96**	**18.96**	**21.89**	**78.11**	**35.01**
**STD**	**1.37**	**3.03**	**4.84**	**0.68**	**3.54**	**3.54**	**2.78**	**2.93**	**6.36**	**1.20**	**5.14**	**7.38**	**1.61**	**6.36**	**6.36**	**5.47**

ENC represents the effective number of codons.

GC1 represents the G + C content at the first position of codons.

GC2 represents the G + C content at the second position of codons.

GC3 represents the G + C content at the third positions of codons.

AU3 represents the A + U content at the third positions of codons.

Nucleotide content analysis at the first, second, and third synonymous codon positions disclosed that the values of GC1 ranged from 28.29 to 41.64% (mean: 34.68%; SD: 2.78), while GC2 ranged from 23.38 to 46.77% (mean: 37.81%; SD: 2.93). However, the GC3 values ranged from 16.28 to 51.64% (mean: 21.89%; SD: 6.36), which is similar to the previous studies on *Taenia pisiformis* (48). In contrast, the values of AU3 ranged from 48.36 to 83.72% (mean: 78.11%; SD: 6.36). These data further supported the notion that an extensive area of *T. saginata* is self-possessed of A/T content (Table 1). This study supports the previous studies on *Taenia solium* and *G. lamblia* (11, 49).

### 3.2. Defining codon usage patterns

**Figure 2:**
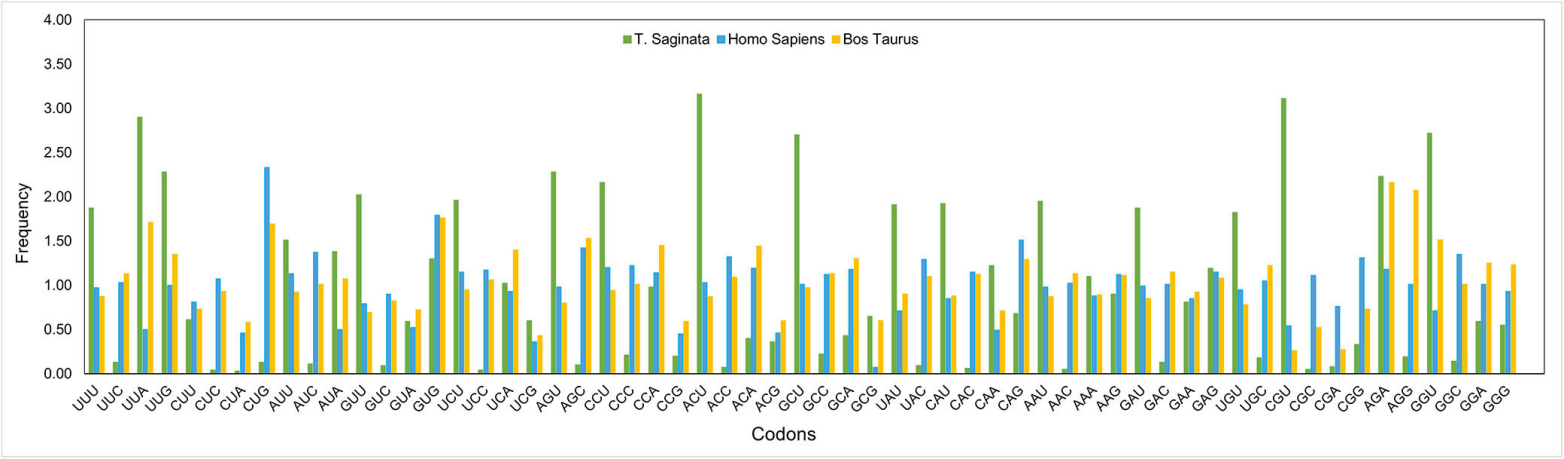
Comparative analysis of RSCU patterns between *Taenia saginata* and its hosts *Bos taurus* and *Homo sapiens*. The *X*-axis represents codons, while the *Y*-axis represents the frequency.

An RSCU analysis was used to regulate the identical pattern of codon usage in the *T. saginata* CDS. Notably, 17 of the 18 most abundantly used codons in *T. saginata* [TTT (Phe), TTA (Leu), ATT (Ile), GTT (Val), AGT (Ser), CCT (Pro), ACT (Thr), GCT (Ala), CAT (His), TAT (Tyr), CAA (Gln), AAT (Asn), AAA (Lys), GAT (Asp), TGT (Cys), CGT (Arg), and GGT (Gly)] ended with T or A (T: 14; A: 3), and the remaining one GAG (Glu) was G ended codons. None of the preferred codons were C-ended. Thus, the A or T-end codon bases are more shared in the genome of *T. saginata*, which is similar to earlier research (45). Furthermore, from the RSCU analysis, we found that the overrepresented (>1.6) codons are rarely seen in the genome of *T. saginata*. Nearly all the ideal and nonideal codons are in the range of 0.6–1.6. We observed that most codons ending in T were overrepresented (>0.6), while codons ending in C were underrepresented (<1.6) (Figure 2, Table 2), revealing that mutational pressure was the primary factor influencing codon usage patterns in *T. saginata*, which was consistent with previous studies (49, 50). From both the nucleotide content and RSCU analysis, we assumed that the selection of the preferred codons has been generally inclined by compositional restraints, which determine the existence of mutational pressure. We are unsure that the compositional pressure could not be the single aspect related to *T. saginata* patterns of codon usage, as although the total values of RSCU could disclose the pattern of codon usage for the genomes, it may conceal the codon usage variation amongst distinct genes in a genome (51).

**Table 2:** The relative synonymous codon usage frequency of *Taenia saginata*, and its natural hosts (*Homo sapiens* and *Bos taurus*).

**AA**	**Codon**	***Taenia saginata***	***Homo sapiens***	***Bos taurus***	**AA**	**Codon**	***Taenia saginata***	***Homo sapiens***	***Bos taurus***
**Phe**	UUU	1.87	0.97	0.87	**His**	CAU	1.92	0.85	0.88
	UUC	0.13	1.03	1.13		CAC	0.06	1.15	1.12
**Leu**	UUA	2.90	0.50	1.71	**Gln**	CAA	1.22	0.49	0.71
	UUG	2.28	1.00	1.35		CAG	0.68	1.51	1.29
	CUU	0.61	0.81	0.73	**Asn**	AAU	1.95	0.98	0.87
	CUC	0.04	1.07	0.93		AAC	0.05	1.02	1.13
	CUA	0.03	0.46	0.58	**Lys**	AAA	1.10	0.88	0.89
	CUG	0.13	2.33	1.69		AAG	0.90	1.12	1.11
**Ile**	AUU	1.51	1.13	0.92	**Asp**	GAU	1.87	0.99	0.85
	AUC	0.11	1.37	1.01		GAC	0.13	1.01	1.15
	AUA	1.38	0.50	1.07	**Glu**	GAA	0.81	0.85	0.92
**Val**	GUU	2.02	0.79	0.69		GAG	1.19	1.15	1.08
	GUC	0.09	0.90	0.82	**Cys**	UGU	1.82	0.95	0.78
	GUA	0.59	0.52	0.72		UGC	0.18	1.05	1.22
	GUG	1.30	1.79	1.76	**Arg**	CGU	3.11	0.54	0.26
**Ser**	UCU	1.96	1.15	0.95		CGC	0.05	1.11	0.52
	UCC	0.04	1.17	1.06		CGA	0.08	0.76	0.27
	UCA	1.02	0.93	1.40		CGG	0.33	1.31	0.73
	UCG	0.60	0.36	0.43		AGA	2.23	1.18	2.16
	AGU	2.28	0.98	0.80		AGG	0.19	1.01	2.07
	AGC	0.10	1.42	1.53	**Gly**	GGU	2.72	0.71	1.51
**Pro**	CCU	2.16	1.20	0.94		GGC	0.14	1.35	1.01
	CCC	0.21	1.22	1.01		GGA	0.59	1.01	1.25
	CCA	0.98	1.14	1.45		GGG	0.55	0.93	1.23
	CCG	0.20	0.45	0.59					
**Thr**	ACU	3.16	1.03	0.87					
	ACC	0.07	1.32	1.09					
	ACA	0.40	1.19	1.44					
	ACG	0.36	0.46	0.60					
**Ala**	GCU	2.70	1.01	0.97					
	GCC	0.22	1.12	1.13					
	GCA	0.43	1.18	1.30					
	GCG	0.65	0.07	0.60					
**Tyr**	UAU	1.91	0.71	0.90					
	UAC	0.09	1.29	1.10					

AA represents amino acid; the “RSCU” value represents the pattern of relative synonymous codon usage; over-represented (RSCU > 1.6), and under-represented (RSCU < 0.6) codons are marked with Red and green, respectively; and the favored codons for Taenia saginata are underlined.

Additionally, to determine whether the CUB of *T. saginata* can be constrained by its hosts (*B. taurus* and *H. sapiens*), all codon RSCU values were also calculated (Table 2). This study indicated that 12 of 59 synonymous codons of *T. saginata* are equivalent to those of *H. sapiens*, individually, and that 16 of 59 synonymous codons are equivalent to those of *B. taurus* (Table 2). In this study, the role of selection from the *B. Taurus* in shaping codon usage patterns of *T. saginata* is different from that of the host *H. sapiens*. It was suggested that the codon usage patterns similarity between *T. saginata* and *B. taurus*/*H. sapiens* can enhance the efficiency of translation in the parasite's genomes (52).

### 3.3. Adaptation of *Taenia saginata* to the host genome

The CAI analysis was executed to regulate the optimization of codon usage and *T. saginata* adaptation to its hosts (53). The values of CAI range from zero to one; a value near one indicates higher levels of CUB (7). For all codons, the CAI values were measured through the reference of *B. taurus* and *H. sapiens* codon usage. We determined that, concerning *B. taurus* and *H. sapiens*, the mean CAI value of *T. saginata* coding regions was 0.59 and 0.68 (>0.5), which revealed that *T. saginata* has a good adaptation to its hosts and a minimal translation pressure (Supplementary Figure S1) (35, 54). The high CAI value tendency of *H. sapiens* recommends that selection pressure from *H. sapiens* should impact the *T. saginata* codon usage and that the codon usage evolution in *T. saginata* should permit it to use the translation machinery of *H. sapiens* more capably. Our result was consistent with published work (55).

To check if the observed significant statistical differences arise in the values of CAI (2, 32), the values of expected CAI (e-CAI) were considered for *T. saginata* CDS with *B. taurus* and *H. sapiens* codon usage sets. The result of the e-CAI value was 0.70 and 0.79 (*P* < 0.05) in relation to *B. taurus* and *H. sapiens*, revealing that the generated sequences keep to a normal distribution. The outcomes of this study about the preferences of codon usage are comparable with previous research (5, 56).

### 3.4. *Bos taurus* has induced stronger selection pressure on *Taenia saginata*

**Figure 3:**
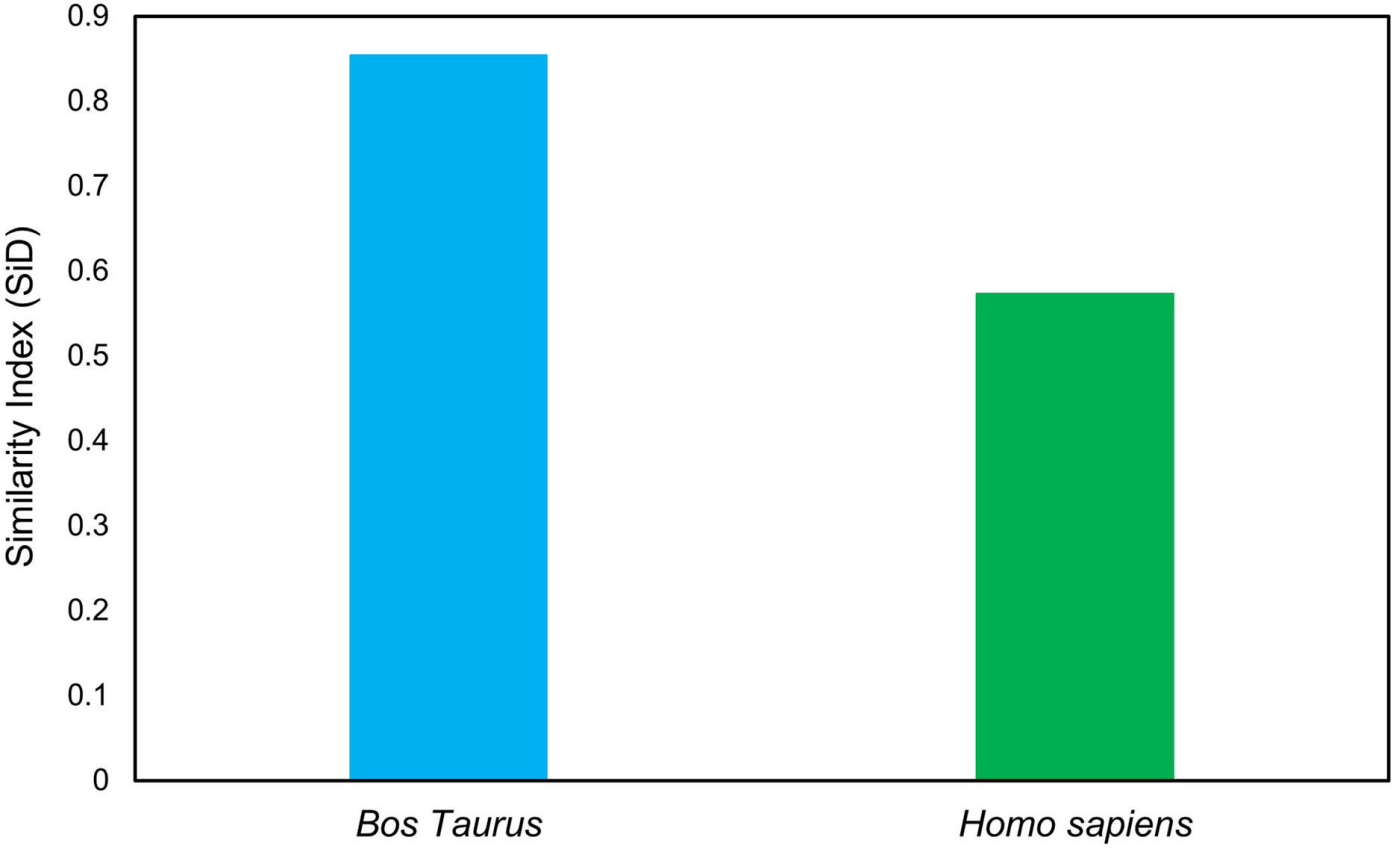
Similarity index analysis of codon usage between *T. saginata's* and its hosts.

The SiD analysis was carried out to assess the potential impact of *B. taurus* and *H. sapiens* codon usage patterns on the evolution of the codon usage patterns of the *T. saginata*. The SiD was found to be higher in *B. taurus*, indicating that it had a more dominant influence on the formation of *T. saginata* codon usage than in *H. sapiens* (Figure 3). Given that *B. taurus* is thought to be the principal reservoir and host of *T. saginata'*s, it is likely that the parasite has stabilized its genetic traits in order to better adapt to the environment of its primary host (33, 57).

### 3.5. Use of codon biases in *Taenia saginata*

To know the strength of CUB within *T. saginata* coding sequences, the gene ENC value was assessed and mapped next to the GC content at the 3rd codon position (GC3; Table 1). In this study, the values of ENC were observed to vary from 33.37 to 58.31, indicating a high level of genetic differences in the codon's usage. Nevertheless, the average value of ENC was 35.02 > 20, implying that the whole CUB was low (Table 2), which was also observed in *T. pisiformis* and Platyhelminthes (48, 50). The analysis disclosed that low codon bias was seen along with the position of natural selection on the genes (43, 58). Therefore, within *T. saginata* coding sequences, low codon bias has permitted *T. saginata* presence in the host, despite the fact that the host maintains codon usage preferences that vary from those of *T. saginata*.

**Figure 4:**
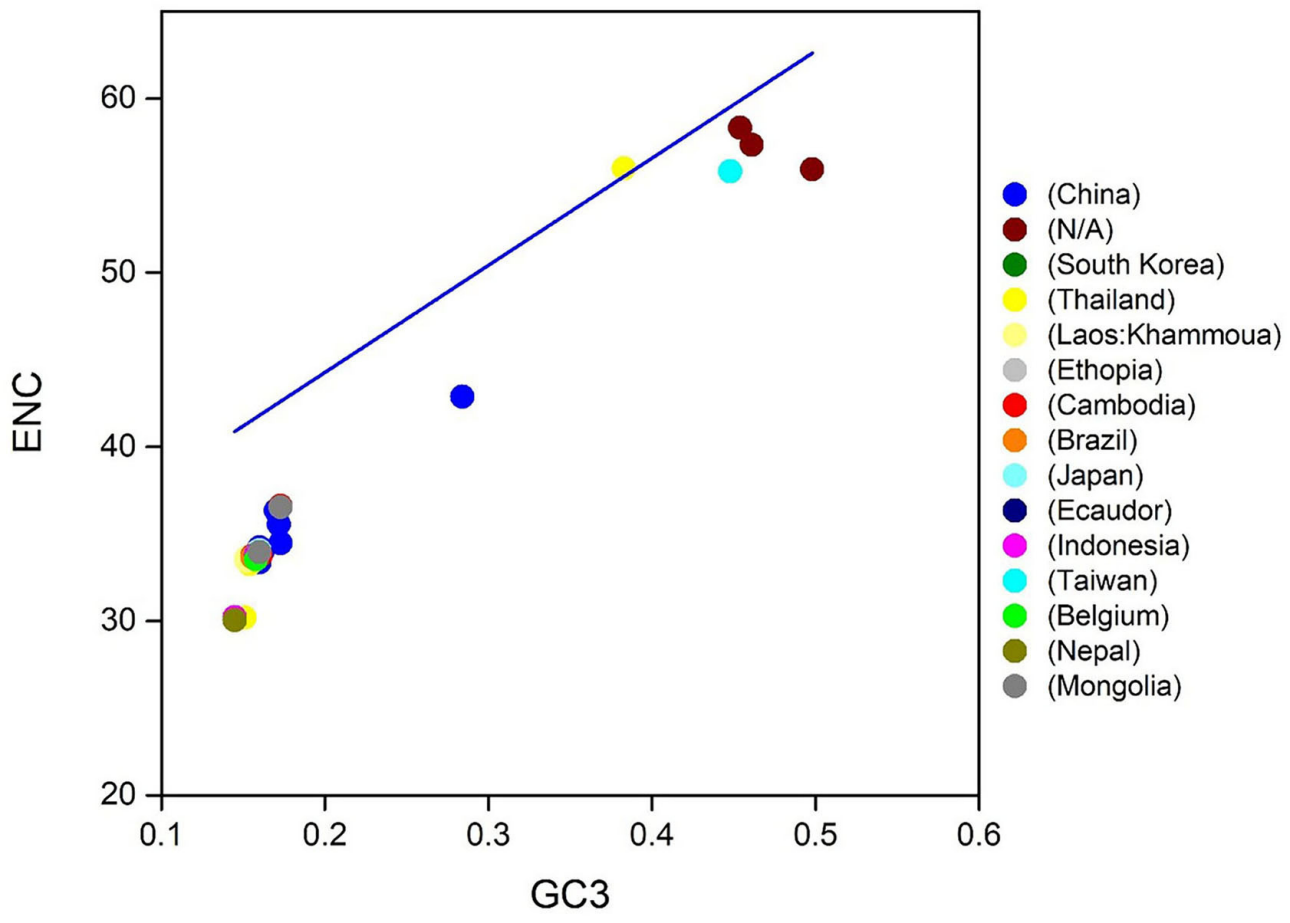
ENC-GC3 plots of 90 *T. saginata* strains: the effective number of codons (ENC-values, *Y*-axis) was plotted against the GC-content at the third synonymous codon positions (GC3-values, *X*-axis).

Then, to determine the codon usage of the genes, a plot of distribution was employed that deviated from the same usage of indistinguishable codons. In this study, ENC values were used against the GC3s. If the GC subject of the gene exhibits mutational pressure, all the points in this plot will be below or close to the expected curve, indicating random codon usage. However, if there is selection pressure on the gene, all the points will lie on or below the expected curve. In this study, we plotted the ENC values of each gene against the GC3 content (Figure 4). The results reveal that mutational pressure and natural selection both influence the codon usage pattern of *T. saginata* genome, as the majority of the points fall below the expected curve and just a few points beyond it (59, 60).

### 3.6. Neutrality plot analysis

**Figure 5:**
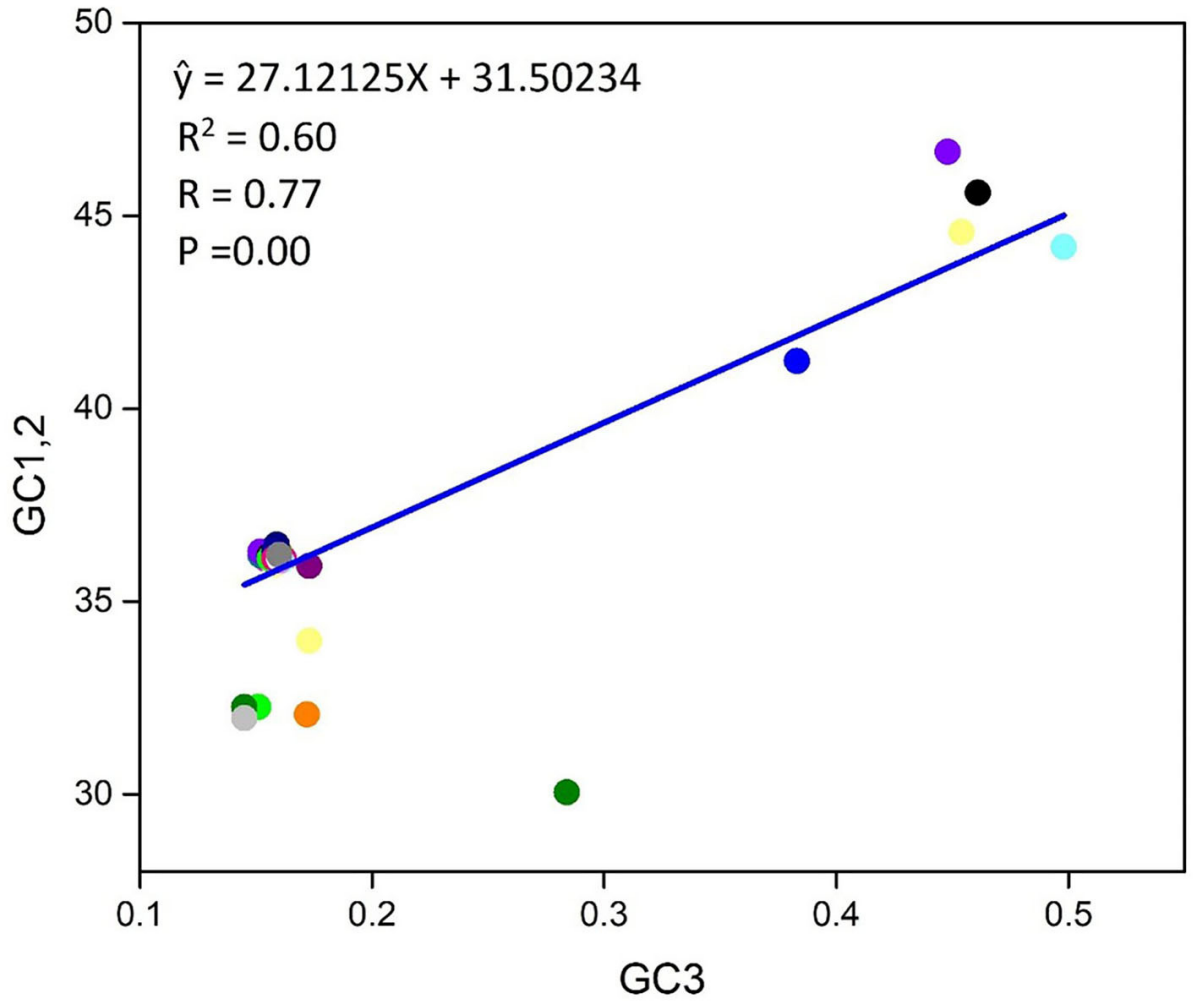
Neutrality plot between (GC3 vs. GC1, 2) for the entire coding sequence of *T. saginata*. GC1, 2 represent GC at the first and second of the codon, while GC3 represents GC at the third codon position. Additionally, the blue solid represents the regression analysis of GC1, 2 against GC3.

A plot of neutrality was performed, which implied the bond between GC1/2 and GC3 composition to determine the position of mutation and selection pressure that has an impact on the CUB form. To observe the association, we programmed a paradigm on the plot of neutrality between GC3 and GC1/2 for the *T. saginata* genome. In this study, the plot shows that no significant association was found between GC3 and GC1/2 contents because the regression value and link are *P* > 0.05 and *r* = −0.77 (Figure 5). Finally, we suggested that both natural selection and mutational pressure have an impact on the codon usage shaping of *T. saginata*. This phenomenon is similar to the previous studies (17, 48, 49).

### 3.7. Discrepancy in the usage of codon among *Taenia saginata*

**Figure 6:**
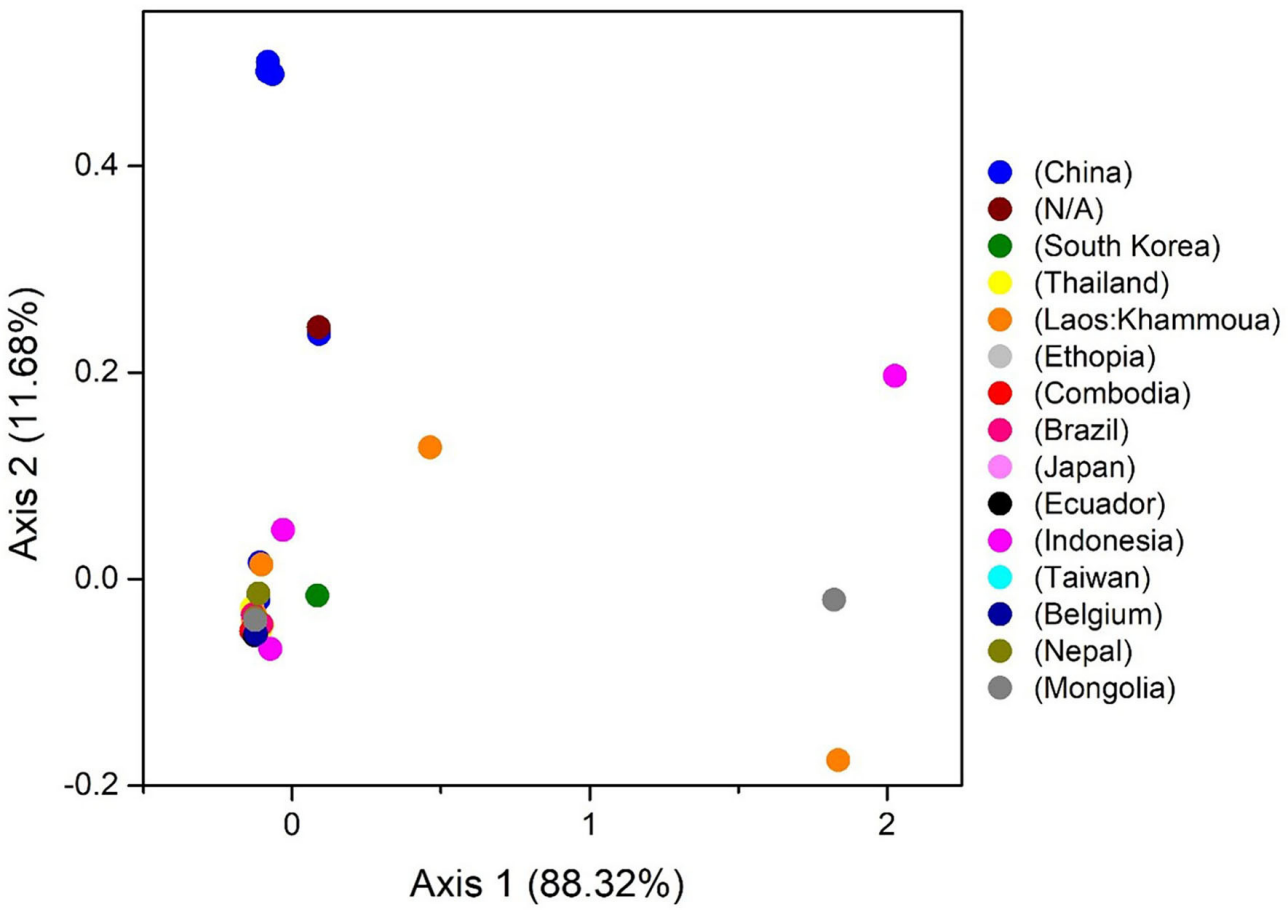
The correspondence analysis (COA) of the genes in *T. saginata* genomes. Each point represents a gene corresponding to the coordinates of the first and second axes of variation generated from the correspondence analysis.

The COA describes the discrepancy in the usage of codons. The changes occur in the patterns of codon usage revealed through RSCU values. In the plot of COA, axis 1 and 2 are the two main factors of general discrepancy (37, 45, 61). We used the values of these two axes to draw COA plots, where each strain is represented by a point, and the distance between strains gives a degree of similarity or dissimilarity in the codon usage patterns. The first and second principal axes accounted for the total variation: 88.32 and 11.68% (Figure 6). These results propose that the first axis signifies the *T. saginata* strains, and the second axis signifies the countries where the *T. saginata* arises. Scattered data on the main axis represents various geographical ancestries and their relationships. All the *T. saginata* strains were found to be in groups using COA. Entire China and all the unknown *T. saginata* strains were assembled into one clade, while *T. saginata* separates from Ethiopia, South Korea, Thailand, Cambodia, Brazil, Ecuador, Taiwan, Belgium, and Nepal were present in the alternative clade. Furthermore, Laos, Indonesia, and Mongolia were divided into separate groups (Figure 6). These studies reveal that the topographical sites play a major part in the evolution of *T. saginata* and in a synonymous codon usage pattern, where in the future, such investigations may assist in discovering the essence of rising *T. saginata* strains. Furthermore, present outcomes also show that more than one widespread genetic lineage was found in every infected country.

**Figure 7:**
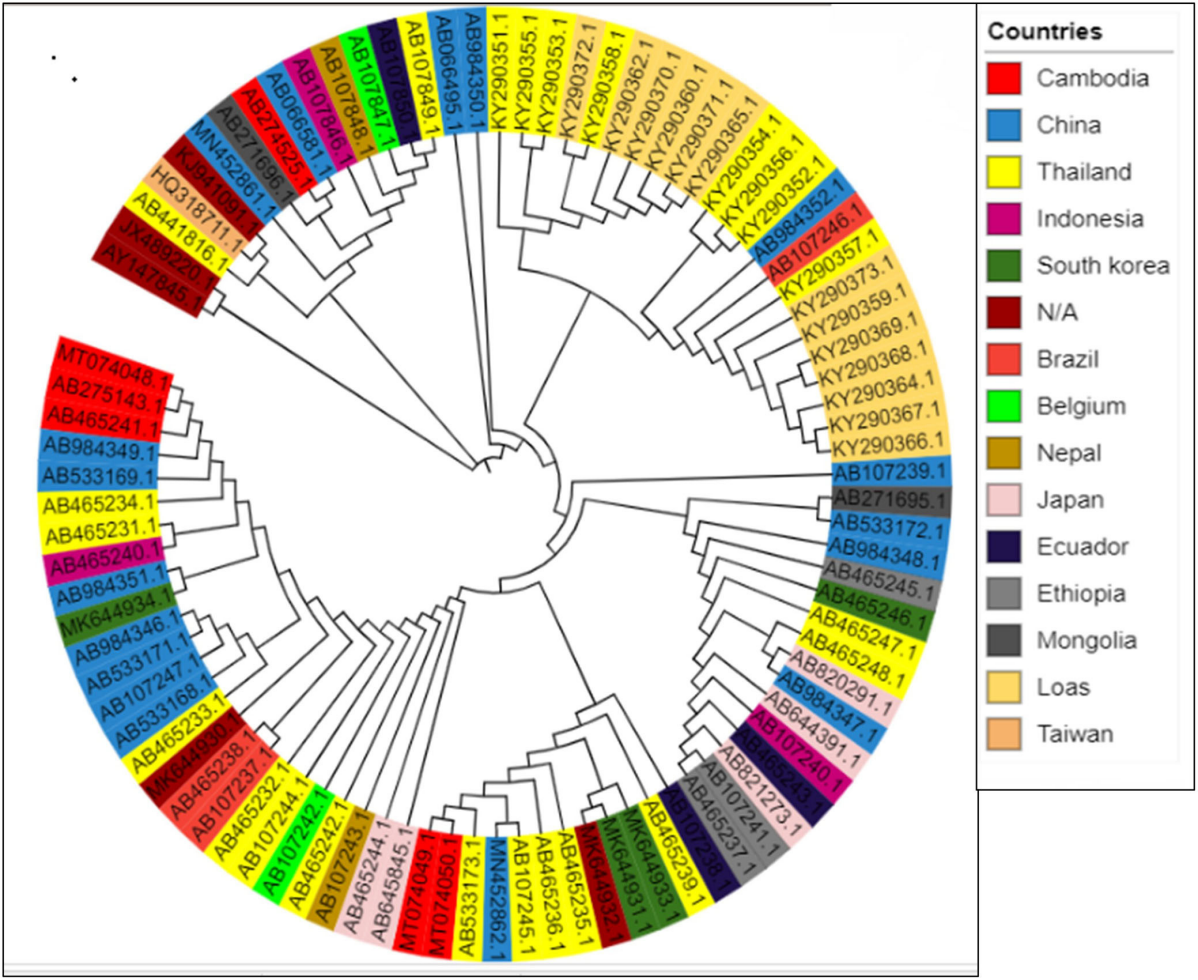
Phylogenetic tree based on the polyprotein-coding regions of 90 *T. saginata* strains. The tree was generated using the maximum likelihood (ML) method using Clustal X2. The tree was designed using the online tool “iTOL.”

To assess the consequence of evolutionary procedures on the *T. saginata* codon usage pattern, a phylogenetic analysis was used through the maximum likelihood method. The entire *T. saginata* separates are dispersed throughout the world, as evidenced by the tree, which shows that no strains form a cluster among different individual countries (Figure 7). The study suggested that this parasite might be altered due to some specific geographical effects such as climatic changes and environmental changes, which support the main outcome of evolutionary processes and topographical dispersal on codon usage patterns. The current study further exposed the signs of recombination and genome reassortment during single-host coinfection, signifying the potential for the upcoming arrival of novel alternates (62, 63).

### 3.8. Codon usage pattern dominating effects on *Taenia saginata*

**Figure 8:**
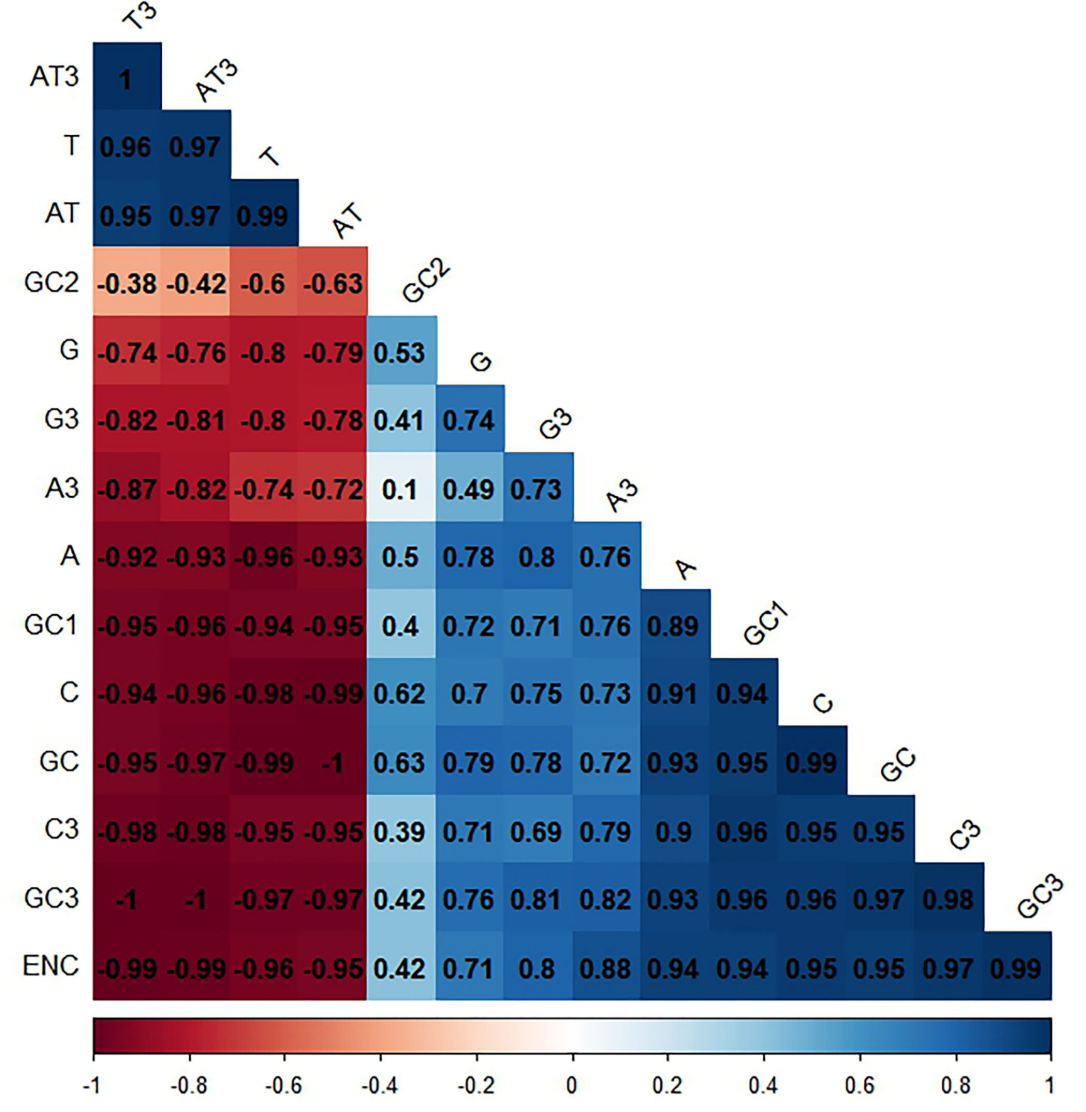
Correlation analysis among different nucleotide contents of *T. saginata*. Dark blue means the positive correlation, and dark red means the negative correlation; the value larger means a more significant correlation.

In this study, we took two factors into consideration: natural selection and mutational pressure, in order to determine CUB in *T. saginata*. Accordingly, we performed a correlation analysis between total nucleotide contents (A, G, C, and T), GC contents (first, second, and third), and ENC. The ENC values of the *T. saginata* sequences seemed to be a positive relationship with GC1, GC2, GC3, A, G, and C except for T, which has a negative relationship that probably affects the *T. saginata* codon usage pattern (Figure 8). Previously, studies suggested that the base compositions at the third position of the codon, mutational bias, are mostly explained, while base compositions at the first and second positions, selective pressure, are mostly validated (64, 65).

Such an impact was also observed among GC, AT, GC3, AT3, A3, C3, G3, and T3 with ENC. The GC, GC3, A3, and C3 have a positive correlation with ENC, whereas the AT, AT3, and T3 have a negative correlation. This result implies the significance of mutational and selection pressure on getting the *T. saginata* codon usage pattern (Figure 8). Additionally, it also suggests that the contents of a nucleotide have an impact on the codon usage pattern of *T. saginata* (66).

## Data availability statement

The original contributions presented in the study are included in the article/Supplementary material, further inquiries can be directed to the corresponding author.

## Author contributions

SR, WM, and DC: conceptualization, methodology, software, data curation, and writing the original draft preparation. IR and HA helped in write-up and editing and validation. HR: methodology, visualization, and validation. MK and FR: reviewing and editing and validation. All authors contributed to the article and approved the submitted version.

## Supplementary material

Supplementary Figure 1 Codon usage adaptation index of *Taenia saginata*'s to its hosts (*Bos taurus* and *Homo sapiens*).

Supplementary Table 1 Detailed information of *Taenia saginata* isolates studied in this study.

## References

[1] Rahman SU Mao Y Tao S. Codon usage bias and evolutionary analyses of Zika virus genomes. Genes and Genomics. (2017) 39:855–66. 10.1007/s13258-017-0549-0

[2] Rahman SU Yao X Li X Chen D Tao S. Analysis of codon usage bias of Crimean-Congo hemorrhagic fever virus and its adaptation to hosts. Infect Genet Evol. (2018) 58:1–16. 10.1016/j.meegid.2017.11.027 29198972

[3] Hershberg R Petrov DA. Selection on codon bias. Annu Rev Genet. (2008) 42:287–99. 10.1146/annurev.genet.42.110807.091442 18983258

[4] Kane JF. Effects of rare codon clusters on high-level expression of heterologous proteins in *Escherichia coli*. Curr Opin Biotechnol. (1995) 6:494–500. 10.1016/0958-1669(95)80082-4 7579660

[5] Yao X Fan Q Yao B Lu P Rahman SU Chen D. Codon usage bias analysis of bluetongue virus causing livestock infection. Front Microbiol. (2020) 11:1–12. 10.3389/fmicb.2020.00655 32508755 PMC7248248

[6] Zheng Y Zhao W-M Wang H Zhou Y-B Luan Y Qi M. Codon usage bias in *Chlamydia trachomatis* and the effect of codon modification in the MOMP gene on immune responses to vaccination. Biochem Cell Biol. (2007) 85:218–26. 10.1139/o06-211 17534403

[7] Gupta SK Bhattacharyya TK Ghosh TC. Synonymous codon usage in *Lactococcus lactis*: mutational bias versus translational selection. J Biomol Struct Dyn. (2004) 21:527–36. 10.1080/07391102.2004.10506946 14692797

[8] Naya H Romero H Carels N Zavala A Musto H. Translational selection shapes codon usage in the GC-rich genome of *Chlamydomonas reinhardtii*. FEBS Lett. (2001) 501:127–30. 10.1016/S0014-5793(01)02644-8 11470270

[9] Lin K Kuang Y Joseph JS Kolatkar PR. Conserved codon composition of ribosomal protein coding genes in *Escherichia coli, Mycobacterium tuberculosis* and *Saccharomyces cerevisiae*: lessons from supervised machine learning in functional genomics. Nucleic Acids Res. (2002) 30:2599–607. 10.1093/nar/30.11.2599 12034849 PMC117187

[10] Kliman RM Irving N Santiago M. Selection conflicts, gene expression, and codon usage trends in yeast. J Mol Evol. (2003) 57:98–109. 10.1007/s00239-003-2459-9 12962310

[11] Lafay B Sharp PM. Synonymous codon usage variation among *Giardia lamblia* genes and isolates. Mol Biol Evol. (1999) 16:1484–95. 10.1093/oxfordjournals.molbev.a026060 10555279

[12] Ghosh TC Gupta SK Majumdar S. Studies on codon usage in *Entamoeba histolytica*. Int J Parasitol. (2000) 30:715–22. 10.1016/S0020-7519(00)00042-4 10856505

[13] Stenico M Lloyd AT Sharp PM. Codon usage *in Caenorhabditis elegans*: delineation of translational selection and mutational biases. Nucleic Acids Res. (1994) 22:2437–46. 10.1093/nar/22.13.2437 8041603 PMC308193

[14] Dermauw V Dorny P Braae UC Devleesschauwer B Robertson LJ Saratsis A. Epidemiology of *Taenia saginata* taeniosis/cysticercosis: a systematic review of the distribution in southern and eastern Africa. Parasit Vectors. (2018) 11:578. 10.1186/s13071-018-3163-3 30400948 PMC6219070

[15] Torgerson PR Abdybekova AM Minbaeva G Shapiyeva Z Thomas LF Dermauw V. Epidemiology of *Taenia saginata* taeniosis/cysticercosis: a systematic review of the distribution in central and western Asia and the Caucasus. Parasit Vectors. (2019) 12:175. 10.1186/s13071-019-3438-3 30999942 PMC6472068

[16] Sapp SGH Bradbury RS. The forgotten exotic tapeworms: a review of uncommon zoonotic Cyclophyllidea. Parasitology. (2020) 147:533–58. 10.1017/S003118202000013X 32048575 PMC7174715

[17] Yang X Luo X Cai X. Analysis of codon usage pattern in *Taenia saginata* based on a transcriptome dataset. Parasites Vectors. (2014) 7:1–11. 10.1186/s13071-014-0527-1 25440955 PMC4268816

[18] Laranjo-González M Devleesschauwer B Gabriël S Dorny P Allepuz A. Epidemiology, impact and control of bovine cysticercosis in Europe: a systematic review. Parasit Vectors. (2016) 9:81. 10.1186/s13071-016-1362-3 26860313 PMC4748494

[19] Trevisan C Sotiraki S Laranjo-González M Dermauw V Wang Z Kärssin A. Epidemiology of taeniosis/cysticercosis in Europe, a systematic review: eastern Europe. Parasit Vectors. (2018) 11:569. 10.1186/s13071-018-3153-5 30376899 PMC6208121

[20] Konyaev S V Nakao M Ito A Lavikainen A. History of *Taenia saginata* tapeworms in Northern Russia. Emerg Infect Dis. (2017) 23:2030–7. 10.3201/eid2312.162101

[21] World Health Organization. WHO/FAO/OIE guidelines for the surveillance, prevention and control of taeniosis/cysticercosis/ editor: Murrell KD; associate editors: Dorny P... [et al.]. (2005). Available online at: https://apps.who.int/iris/handle/10665/43291 (accessed August 01, 2022).

[22] Craig P Ito A. Intestinal cestodes. Curr Opin Infect Dis. (2007) 20:524–32. 10.1097/QCO.0b013e3282ef579e 17762788

[23] Egger B. Making heads or tails of tapeworms. Trends Parasitol. (2016) 32:511–2. 10.1016/j.pt.2016.04.003 27095055

[24] Ríos-Valencia DG. To be or not to be a tapeworm parasite: that is the post-genomic question in *Taenia solium* cysticercosis. In:Navarrete-Perea J, editor. Current State of the Art in Cysticercosis and Neurocysticercosis. Rijeka: IntechOpen. (2021), p. 107. 10.5772/intechopen.97306

[25] Bordon LM. Intestinal obstruction due to *Taenia saginata* infection: a case report. J Trop Med Hyg. (1992) 95:352–3. 1404561

[26] Silva C V Costa-Cruz JM. A glance at *Taenia saginata* infection, diagnosis, vaccine, biological control and treatment. Infect Disord Drug Targets. (2010) 10:313–21. 10.2174/187152610793180894 20701576

[27] Karumathil S Raveendran NT Ganesh D Kumar Ns S Nair RR Dirisala VR. Evolution of synonymous codon usage bias in West African and Central African strains of Monkeypox virus. Evol Bioinform Online. (2018) 14:1176934318761368. 10.1177/1176934318761368 29551886 PMC5846927

[28] Sharp PM Li WH. Codon usage in regulatory genes in *Escherichia coli* does not reflect selection for “rare” codons. Nucleic Acids Res. (1986) 14:7737–49. 10.1093/nar/14.19.7737 3534792 PMC311793

[29] Wong EHM Smith DK Rabadan R Peiris M Poon LLM. Codon usage bias and the evolution of influenza A viruses. Codon usage biases of influenza virus. BMC Evol Biol. (2010) 10:253. 10.1186/1471-2148-10-253 20723216 PMC2933640

[30] Liu J Zhu D Ma G Liu M Wang M Jia R. Genome-wide analysis of the synonymous codon usage patterns in *Riemerella anatipestifer*. Int J Mol Sci. (2016) 17:1304. 10.3390/ijms17081304 27517915 PMC5000701

[31] Chakraborty A Uechi T Higa S Torihara H Kenmochi N. Loss of ribosomal protein L11 affects zebrafish embryonic development through a p53-dependent apoptotic response. PLoS ONE. (2009) 4:e4152. 10.1371/journal.pone.0004152 19129914 PMC2612748

[32] Puigbò P Bravo IG Garcia-Vallve S. CAIcal: a combined set of tools to assess codon usage adaptation. Biol Direct. (2008) 3:38. 10.1186/1745-6150-3-38 18796141 PMC2553769

[33] Butt AM Nasrullah I Qamar R Tong Y. Evolution of codon usage in Zika virus genomes is host and vector specific. Nat Publ Gr. (2016) 5:e107. 10.1038/emi.2016.106 27729643 PMC5117728

[34] Wright F. The “effective number of codons” used in a gene. Gene. (1990) 87:23–9. 10.1016/0378-1119(90)90491-9 2110097

[35] Rahman SU Abdullah M Khan AW Haq MIU Haq NU Aziz A Tao S. A detailed comparative analysis of codon usage bias in Alongshan virus. Virus Res. (2022) 308:198646. 10.1016/j.virusres.2021.198646 34822954

[36] Comeron JM Aguade M. An evaluation of measures of synonymous codon usage bias. J Mol Evol. (1998) 47:268–74. 10.1007/PL00006384 9732453

[37] Greenacre M. Theory and Applications of Correspondence Analysis. Cambridge, MA: Academic Press (1984).

[38] Cristina J Fajardo A Sonora M Moratorio G Musto H. A detailed comparative analysis of codon usage bias in Zika virus. Virus Res. (2016) 223:147–52. 10.1016/j.virusres.2016.06.022 27449601

[39] Letunic I Bork P. Interactive tree of life v2: online annotation and display of phylogenetic trees made easy. Nucleic Acids Res. (2011) 39:475–8. 10.1093/nar/gkr201 21470960 PMC3125724

[40] Serres-Giardi L Belkhir K David J Glemin S. Patterns and evolution of nucleotide landscapes in seed plants. Plant Cell. (2012) 24:1379–97. 10.1105/tpc.111.093674 22492812 PMC3398553

[41] Guan DL Ma L Khan MS Zhang XX Xu SQ Xie JY. Analysis of codon usage patterns in *Hirudinaria manillensis* reveals a preference for GC-ending codons caused by dominant selection constraints. BMC Genomics. (2018) 19:1–14. 10.1186/s12864-018-4937-x 30016953 PMC6050667

[42] Sueoka N. Directional mutation pressure and neutral molecular evolution. Proc Natl Acad Sci U S A. (1988) 85:2653–7. 10.1073/pnas.85.8.2653 3357886 PMC280056

[43] Wu Y Zhao D Tao J. Analysis of codon usage patterns in herbaceous peony (*Paeonia lactiflora* Pall) based on transcriptome data. Genes. (2015) 6:1125–39. 10.3390/genes6041125 26506393 PMC4690031

[44] Singh NK Tyagi A. A detailed analysis of codon usage patterns and influencing factors in Zika virus. Arch Virol. (2017) 162:1963–73. 10.1007/s00705-017-3324-2 28324177

[45] Butt AM Nasrullah I Tong Y. Genome-wide analysis of codon usage and influencing factors in chikungunya viruses. PLoS ONE. (2014) 9:e0090905. 10.1371/journal.pone.0090905 24595095 PMC3942501

[46] Waterkeyn JG Gauci C Cowman AF Lightowlers MW. Codon usage in *Taenia* species. Exp Parasitol. (1998) 88:76–8. 10.1006/expr.1998.4215 9501853

[47] van Hemert F Berkhout B. Nucleotide composition of the Zika virus RNA genome and its codon usage. Virol J. (2016) 13:95. 10.1186/s12985-016-0551-1 27278486 PMC4898363

[48] Chen L Liu T Yang D Nong X Xie Y Fu Y. Analysis of codon usage patterns in *Taenia pisiformis* through annotated transcriptome data. Biochem Biophys Res Commun. (2013) 430:1344–8. 10.1016/j.bbrc.2012.12.078 23268345

[49] Yang X Ma X Luo X Ling H Zhang X Cai X. Codon usage bias and determining forces in *Taenia solium* genome. Korean J Parasitol. (2015) 53:689–97. 10.3347/kjp.2015.53.6.689 26797435 PMC4725240

[50] Le TH McManus DP Blair D. Codon usage and bias in mitochondrial genomes of parasitic platyhelminthes. Korean J Parasitol. (2004) 42:159–67. 10.3347/kjp.2004.42.4.159 15591833 PMC2717381

[51] Hassan S Mahalingam V Kumar V. Synonymous codon usage analysis of thirty two mycobacteriophage genomes. Adv Bioinformatics. (2009) 2009:316936. 10.1155/2009/316936 20150956 PMC2817497

[52] Ma X-X Feng Y-P Bai J-L Zhang D-R Lin X-S Ma Z-R. Nucleotide composition bias and codon usage trends of gene populations in *Mycoplasma capricolum* subsp. capricolum and *M agalactiae. J Genet*. (2015) 94:251–60. 10.1007/s12041-015-0512-2 26174672

[53] Sharp PM Li WH. An evolutionary perspective on synonymous codon usage in unicellular organisms. J Mol Evol. (1986) 24:28–38. 10.1007/BF02099948 3104616

[54] Subramanian A Rup Sarkar R. Data in support of large scale comparative codon usage analysis in Leishmania and Trypanosomatids. Data Br. (2015) 4:269–72. 10.1016/j.dib.2015.06.003 26217801 PMC4510541

[55] Pan X Welti R Wang X. Quantitative analysis of major plant hormones in crude plant extracts by high-performance liquid chromatography–mass spectrometry. Nat Protoc. (2010) 5:986–92. 10.1038/nprot.2010.37 20448544

[56] Maldonado LL Stegmayer G Milone DH Oliveira G Rosenzvit M Kamenetzky L. Whole genome analysis of codon usage in *Echinococcus*. Mol Biochem Parasitol. (2018) 225:54–66. 10.1016/j.molbiopara.2018.08.001 30081061

[57] Chantawannakul P Cutler RW. Convergent host–parasite codon usage between honeybee and bee associated viral genomes. J Invertebr Pathol. (2008) 98:206–10. 10.1016/j.jip.2008.02.016 18397791

[58] Ma YP Liu ZX Hao L Ma JY Liang ZL Li YG Ke H. Analysing codon usage bias of cyprinid herpesvirus 3 and adaptation of this virus to the hosts. J Fish Dis. (2015) 38:665–73. 10.1111/jfd.12316 25491502

[59] Li X Wang X Gong P Zhang N Zhang X Li J. Analysis of codon usage patterns in giardia duodenalis based on transcriptome data from GiardiaDB. Genes. (2021) 12:1169. 10.3390/genes12081169 34440343 PMC8393687

[60] Zhou J-H Gao Z-L Zhang J Ding Y-Z Stipkovits L Szathmary S. The analysis of codon bias of foot-and-mouth disease virus and the adaptation of this virus to the hosts. Infect Genet Evol J Mol Epidemiol Evol Genet Infect Dis. (2013) 14:105–10. 10.1016/j.meegid.2012.09.020 23220329

[61] Kumar N Bera BC Greenbaum BD Bhatia S Sood R Selvaraj P. Revelation of influencing factors in overall codon usage bias of equine influenza viruses. PLoS ONE. (2016) 11:e0154376. 10.1371/journal.pone.0154376 27119730 PMC4847779

[62] Chamberlain J Cook N Lloyd G Mioulet V Tolley H Hewson R. Co-evolutionary patterns of variation in small and large RNA segments of Crimean-Congo hemorrhagic fever virus. J Gen Virol. (2005) 86:3337–41. 10.1099/vir.0.81213-0 16298979

[63] Hewson R Gmyl A Gmyl L Smirnova SE Karganova G Jamil B. Evidence of segment reassortment in Crimean-Congo haemorrhagic fever virus. J Gen Virol. (2004) 85:3059–70. 10.1099/vir.0.80121-0 15448369

[64] RoyChoudhury S Mukherjee D. A detailed comparative analysis on the overall codon usage pattern in herpesviruses. Virus Res. (2010) 148:31–43. 10.1016/j.virusres.2009.11.018 19969032

[65] Hu C Chen J Ye L Chen R Zhang L Xue X. Codon usage bias in human cytomegalovirus and its biological implication. Gene. (2014) 545:5–14. 10.1016/j.gene.2014.05.018 24814188

[66] Chen H Sun S Norenburg JL Sundberg P. Mutation and selection cause codon usage and bias in mitochondrial genomes of ribbon worms (Nemertea). PLoS ONE. (2014) 9:e0085631. 10.1371/journal.pone.0085631 24454907 PMC3893253

