# Supplementary figures and images for: Evolution of codon usage in Taenia saginata genomes and its impact on the host
Source: Front Vet Sci. 2023 Jan 11;9:1021440. doi: 10.3389/fvets.2022.1021440 (PMC9875090; doi:10.3389/fvets.2022.1021440)

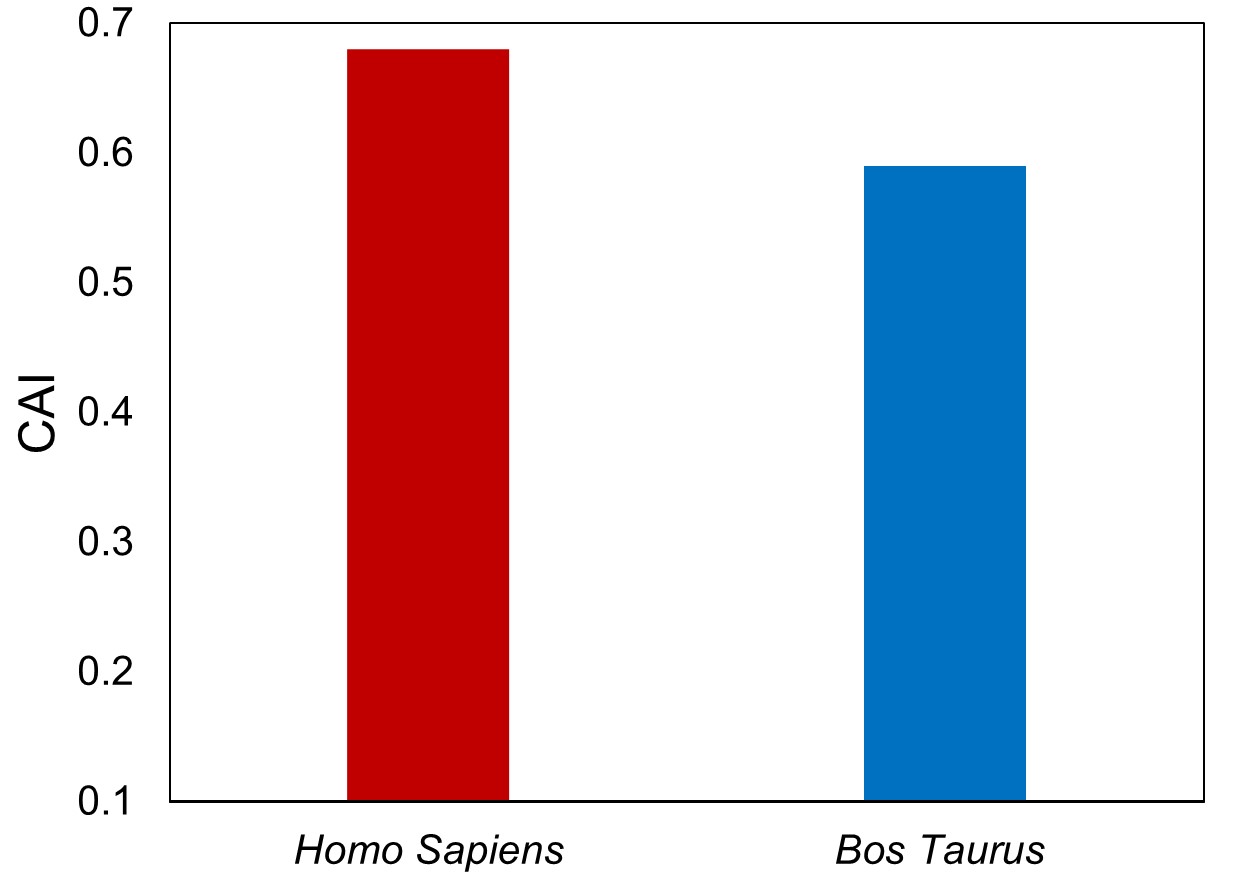

Supplement: Supplementary Figure 1 — Codon usage adaptation index of Taenia saginata's to its hosts (Bos taurus and Homo sapiens). [file Image_1.JPEG]
