# Supplementary material for: Evolution of codon usage in Taenia saginata genomes and its impact on the host
Source: Front Vet Sci. 2023 Jan 11;9:1021440. doi: 10.3389/fvets.2022.1021440 (PMC9875090; doi:10.3389/fvets.2022.1021440)
Supplement: Supplementary Table 1 — Detailed information of Taenia saginata isolates studied in this study. [file Table_1.DOCX]

**Table S1:** Detailed information of *Taenia Saginata* isolates studied in this study.

| S. No | Accession No | Country | Sequence length |
| --- | --- | --- | --- |
|  | JX489220.1 | N\A | 2982 bp |
|  | AB984346.1 | China | 1620 bp |
|  | AB984347.1 | China | 1620 bp |
|  | AB984348.1 | China | 1620 bp |
|  | AB984349.1 | China | 1620 bp |
|  | AB984350.1 | China | 1620 bp |
|  | AB984351.1 | China | 1620 bp |
|  | AB984352.1 | China | 1620 bp |
|  | KJ941091.1 | N\A | 825 bp |
|  | MK644930.1 | N\A | 1620 bp |
|  | MK644932.1 | N\A | 1620 bp |
|  | MK644933.1 | South Korea | 1620 bp |
|  | MK644934.1 | South Korea | 1620 bp |
|  | AB465231.1 | Thailand | 1620 bp |
|  | AB465232.1 | Thailand | 1620 bp |
|  | AB465233.1 | Thailand | 1620 bp |
|  | AB465234.1 | Thailand | 1620 bp |
|  | AB465235.1 | Thailand | 1620 bp |
|  | AB465236.1 | Thailand | 1620 bp |
|  | AB465237.1 | Ethiopia | 1620 bp |
|  | AB465238.1 | Brazil | 1620 bp |
|  | AB465239.1 | Thailand | 1620 bp |
|  | AB465240.1 | Indonesia | 1620 bp |
|  | AB465241.1 | Cambodia | 1620 bp |
|  | AB465242.1 | Thailand | 1620 bp |
|  | AB465243.1 | Ecuador | 1620 bp |
|  | AB465244.1 | Japan | 1620 bp |
|  | AB465245.1 | Ethiopia | 1620 bp |
|  | AB465246.1 | South Korea | 1620 bp |
|  | AB465247.1 | Thailand | 1620 bp |
|  | AB465248.1 | Thailand | 1620 bp |
|  | AB533168.1 | China | 1620 bp |
|  | AB533169.1 | China | 1620 bp |
|  | AB533171.1 | China | 1620 bp |
|  | AB533172.1 | China | 1620 bp |
|  | AB533173.1 | Thailand | 1620 bp |
|  | HQ318711.1 | Taiwan | 1392 bp |
|  | AB645845.1 | Japan | 1620 bp |
|  | AB644391.1 | Japan | 1620 bp |
|  | AB821273.1 | Japan | 1620 bp |
|  | AB820291.1 | Japan | 1620 bp |
|  | KY290351.1 | Thailand | 1620 bp |
|  | KY290352.1 | Thailand | 1620 bp |
|  | KY290353.1 | Thailand | 1620 bp |
|  | KY290354.1 | Thailand | 1620 bp |
|  | KY290355.1 | Thailand | 1620 bp |
|  | KY290356.1 | Thailand | 1620 bp |
|  | KY290357.1 | Thailand | 1620 bp |
|  | KY290358.1 | Thailand | 1620 bp |
|  | KY290359.1 | Laos | 1620 bp |
|  | KY290360.1 | Laos | 1620 bp |
|  | KY290362.1 | Laos | 1620 bp |
|  | KY290364.1 | Laos | 1620 bp |
|  | KY290365.1 | Laos | 1620 bp |
|  | KY290366.1 | Laos | 1620 bp |
|  | KY290367.1 | Laos | 1620 bp |
|  | KY290368.1 | Laos | 1620 bp |
|  | KY290369.1 | Laos | 1620 bp |
|  | KY290370.1 | Laos | 1620 bp |
|  | KY290371.1 | Laos | 1620 bp |
|  | KY290372.1 | Laos | 1620 bp |
|  | KY290373.1 | Laos | 1620 bp |
|  | MK644931.1 | South Korea | 1620 bp |
|  | MT074048.1 | Cambodia | 1635 bp |
|  | MT074049.1 | Cambodia | 1635 bp |
|  | MT074050.1 | Cambodia | 1635 bp |
|  | MN452861.1 | China | 912 bp |
|  | MN452862.1 | China | 1620 bp |
|  | AB066495.1 | China | 1620 bp |
|  | AB066581.1 | China | 1068 bp |
|  | AB107237.1 | Brazil | 1620 bp |
|  | AB107238.1 | Ecuador | 1620 bp |
|  | AB107239.1 | China | 1620 bp |
|  | AB107240.1 | Indonesia | 1620 bp |
|  | AB107241.1 | Ethiopia | 1620 bp |
|  | AB107242.1 | Belgium | 1620 bp |
|  | AB107243.1 | Nepal | 1620 bp |
|  | AB107244.1 | Thailand | 1620 bp |
|  | AB107245.1 | Thailand | 1620 bp |
|  | AB107246.1 | Brazil | 1620 bp |
|  | AB107247.1 | Chin | 1620 bp |
|  | AB274525.1 | Cambodia | 1068 bp |
|  | AB275143.1 | Cambodia | 1068 bp |
|  | AB271695.1 | Mongolia | 1068 bp |
|  | AB271696.1 | Mongolia | 1068 bp |
|  | AB107846.1 | Indonesia | 516 bp |
|  | AB107847.1 | Brazil | 516 bp |
|  | AB107848.1 | Nepal | 516 bp |
|  | AB107849.1 | Thailand | 516 bp |
|  | AB441816.1 | Thailand | 3165 bp |
